# Exploring the relationship between unemployment perception and health during COVID-19: a comparative study of rural and urban adults in China

**DOI:** 10.3389/fpsyg.2023.1169845

**Published:** 2023-06-14

**Authors:** Fengtian Zheng, Huanhuan Xiong, Yanhong Jin, Man Zhang

**Affiliations:** ^1^School of Agricultural Economics and Rural Development, Renmin University of China, Beijing, China; ^2^Department of Agricultural, Food and Resource Economics, School of Environmental and Biological Sciences, Rutgers, The State University of New Jersey, New Brunswick, NJ, United States

**Keywords:** unemployment perception, psychological wellbeing, health, rural and urban adults, China

## Abstract

**Introduction:**

The COVID-19 pandemic has significantly impacted global economies and individual health. This study uses data from the China Family Panel Data (CFPS) in 2018 (before the pandemic) and 2020 (during the pandemic) to a) estimate the relationships between the perceived risk of unemployment and individuals' mental health, physical health, and health behaviors; and b) explore the variations of these relationships between rural and urban adults in China.

**Methods:**

Ordinary linear regression models or Logit models are employed, depending on the nature (continuous vs. discrete) of the dependent variables.

**Results:**

We find that the perceived risk of unemployment was statistically and positively associated with the risk of depression, and the association was greater for rural adults than for urban adults. Rural-urban variations were observed in various dimensions. For rural adults, the perceived risk of unemployment was statistically and negatively associated with life satisfaction, the probability of gaining weight and becoming obese, the probability of having adequate sleep, and computer-based screen time. These associations were statistically insignificant for urban adults. On the other hand, the perceived risk of unemployment was statistically and negatively associated with self-rated very-good-to-excellent health condition and health-compromising behaviors (e.g., smoking and drinking) for urban adults; but such associations were statistically insignificant for rural adults.

**Discussion:**

These findings suggest that rural and urban adults exhibited different psychological and behavioral responses to the unemployment risk during the COVID-19 pandemic. Public policies aiming to improve health and employment should be strategically designed to address the unique needs of urban and rural populations.

## Introduction

The COVID-19 pandemic has been an ongoing global public health crisis, causing more than 600 million confirmed infection cases with over 6 million deaths worldwide (WHO, 2023). Governments have implemented various policies, including social distancing, isolations, travel restrictions, lockdowns, and masking mandates, to curtain the spread. The pandemic has seriously affected the world's economic and political conditions (Chen G. et al., 2022; Fang et al., 2022a,b). The economic impacts have been globally unprecedented—more than 90% of countries worldwide had a negative GDP growth in 2020, a much higher percentage than that in World War I and II as well as the 2008 economic recession (World-Bank, 2022). Economic downturns or even recessions increased unemployment worldwide (Che et al., 2020; Galea and Abdalla, 2020; Su et al., 2022) and led to a poor employment outlook (Aucejo et al., 2020; Le and Nguyen, 2021).

The relationships between economic condition and health outcomes vary by the type and severity of economic slump, cultural backgrounds, geographic aggregation, and population groups (Ruhm, 2000, 2005; Janet et al., 2015; Alam and Bose, 2022; Belotti et al., 2022). Some studies find that higher unemployment rates during business cycle fluctuations are associated with better health outcomes, such as lower mortality risk, reduced smoking, and decreased obesity prevalence. This association is attributed to the decreased opportunity cost of time, which encourages people to invest more time in maintaining and improving their health (Ruhm, 2000, 2005). Others find that sharp and severe economic shocks, such as the 2008 economic recession, can have detrimental effect on health outcomes. These effects may include a decline in self-reported health status, an increase in substance use, and a higher prevalence of cardiovascular diseases-related morbidity. (Janet et al., 2015; De and Segura-Escano, 2021; Belotti et al., 2022).

Unlike economic downturns and recessions in the past, the economic downturn during the COVID-19 pandemic was caused by an unprecedented public health crisis. Job insecurity along with economic downturns and health shocks is expected to affect mental health and psychological wellbeing during the pandemic (Ganson et al., 2021; Gong et al., 2022; Sun et al., 2022). However, very few studies have provided comprehensive evaluations on how individuals' psychological wellbeing, physical health, and health behaviors were affected by job insecurity during the pandemic.

Since the first infected case was reported in December 2019, China has implemented strict lockdown measures such as the “zero-COVID” policy through extensive testing, contact tracing, and quarantine apparatus (Normile, 2021; Wang et al., 2021). Several studies have found that the pandemic elevated the level of stress and anxiety across different age cohorts in China (Hu et al., 2021; Chen B. et al., 2022; Yang et al., 2022). On the other hand, although China had the lowest GDP growth rate^1^ (2.2%) and the highest unemployment rate^2^ (4.2%) in 2020 in the last decade, it was one of the very few countries having positive GDP growth during the pandemic (World-Bank, 2022). Yet, no study has investigated the relationships between perceived job insecurity and a broad spectrum of health status among Chinese adults.

In China, rural–urban disparities and inequalities have been predominant in many socioeconomic perspectives, such as income, infrastructure, health services, fertility, mortality, education, and social security system (Goodkind, 2011; Xie and Zhou, 2014; Bragg et al., 2017; Chan and Wei, 2019; Che et al., 2020). The rural populations, especially rural-to-urban migrant workers, were more vulnerable to economic shocks during the pandemic (Che et al., 2020; Wang et al., 2021). However, no studies have explored the potential differences in the relationship between the perceived risk of unemployment and health outcomes/behaviors between urban and rural adults in China. This study fills the gap in literature by exploring the rural–urban variations in such relationships.

Using the China Family Panel Data (CFPS) in 2018 (before the pandemic) and 2020 (during the pandemic), we find that a higher perceived risk of unemployment was found to be statistically associated with lower life satisfaction and an increased risk of depression. This is evidence of rural–urban disparities in the relationships between employment risk and health outcomes from various perspectives. The association of unemployment risk with life satisfaction was found to be statistically significant and negative for rural adults but statistically insignificant for urban adults. Similar patterns were found for health outcomes (whether gained weight or became obese) and health behaviors (computer-based screen times and having adequate sleep). On the other hand, the association between unemployment risk and health outcomes (self-reported very-good/excellent health condition) and health-compromising behaviors (smoking and drinking) were found to be statistically significant and negative for urban adults, but statistically insignificant for rural adults.

This study expands the emerging literature by investigating the relationships between unemployment risk triggered by an unprecedented health crisis and individual health outcomes, which differs from the previous studies focusing on economic downturns in business fluctuation cycles or by economic shocks (e.g., the great recession in 2008) (Ruhm, 2000, 2005; Burgard and Kalousova, 2015; Janet et al., 2015; Alam and Bose, 2022). Our empirical results based on nationally representative data show that Chinese adults who perceived their job as insecure were less satisfied with their lives and more likely to experience depression. Rural and urban adults might respond to and cope with job insecurity differently and thus experience differentiated health impacts—decreased probability of gaining weight and less computer-based screen time for rural adults and reduced smoking and drinking for urban adults.

This study contributes to the literature on social inequality. Previous studies show that individuals with low income or socioeconomic status, limited skills, poor health, or limited access to healthcare are more susceptible to unemployment and more likely to experience adverse mental health outcomes during the COVID-19 pandemic (Banks et al., 2020; Che et al., 2020; Gong et al., 2022). Our study is the first to present rural–urban variations on comprehensive health outcomes and behaviors affected by unemployment risk during the pandemic for Chinese adults. Our findings suggest that public policies aimed to improve health outcomes and behaviors impacted by job insecurity during the COVID-19 pandemic should be tailored to target urban and rural populations specifically.

## Literature overview of economic downturns and health outcomes

### Economic downturns and health outcomes

How could macroeconomic conditions affect individual health outcomes? The theoretical foundation can be traced back to Grossman (1972)'s seminal work on health production modeling. An economic downturn potentially leads to unemployment, income loss, and wage decrease, affecting an individual's resource reallocation for health production. On the one hand, a wage decrease leads to a lower opportunity cost of time. Therefore, individuals would increase time spent in health-promising activities (e.g., more physical activity and cooking healthier food) due to the substitution effect. On the other hand, due to the income effect, job loss and income/wage decrease make people less affordable for healthy foods and health-promising activities. Furthermore, people may seek health-comprising activities, such as substance use, to deal with increased stress or anxiety resulting from adverse labor market outcomes.

Since the substitution and income effects on health outcomes are in opposite directions, whether the net impact is positive or not is an empirical issue. Ruhm et al. provide empirical evidence supporting the “healthy life in hard times” hypothesis (Ruhm, 2000, 2003, 2005; Ruhm and Black, 2002; Gerdtham and Ruhm, 2006). They find pro-cyclical associations between unemployment and healthy lifestyles: a higher unemployment rate is associated with a lower probability of mortality, obesity, and acute and chronic illness (Ruhm, 2000, 2003, 2005; Ruhm and Black, 2002; Gerdtham and Ruhm, 2006). Others find that job insecurity leads to poor health outcomes (Charles and DeCicca, 2008; Browning and Heinesen, 2012; Cygan-Rehm et al., 2017; Lenhart, 2017). A higher unemployment rate correlates with weight gain (Böckerman et al., 2007; Latif, 2014), increased tobacco and alcohol consumption (Dávalos et al., 2012), and higher morbidity of cardiovascular diseases (Belotti et al., 2022). Given that the relationship between economic downturns and health outcomes is an empirical issue depending on the trade-off between the substitution and income effects, further research is needed in various contexts and among different population groups.

### COVID-19 pandemic and health outcomes

The COVID-19 pandemic has significantly changed people's lifestyles and psychological wellbeing. Blanchflower and Bryson (2022) report that anxiety and depression for U.S. adults reached a peak in 2020 and then declined in 2021 and 2022 using the Household Pulse Survey data collected by the U.S. Census Bureau. A meta-analysis based on 71 studies covering 146,139 adults, including Chinese, Japanese, Italian, American, Turkish, Indian, Spanish, Greek, and Singaporean populations, shows that many people suffered from anxiety (32.6%), depression (27.6%), and insomnia (30.3%) during the pandemic (Liu et al., 2021). The elderly, low-income households, and ethnic minorities were more vulnerable to the pandemic as they experienced poor mental health and higher mortality (Proto and Quintana-Domeque, 2021; Whitehead et al., 2021).

The COVID-19 pandemic also led to changes in individual health-promising and compromising behaviors. Individuals may increase substance use to cope with elevated anxiety or depression (Rehm et al., 2020; Rodriguez et al., 2021). Bremner (2020) finds that sales of alcoholic beverages in the U.S. increased by 55% in the week ending 28 March 2020, compared with the same week in 2019. Increased alcohol consumption during the pandemic was also found in Canada (NANOS-Research, 2020). Two studies for England population during the lockdown offer mixed results: Jackson et al. (2021) find reduced alcohol consumption using the monthly cross-sectional survey among the adult population, while Kim et al. (2020) report increased alcohol consumption among people with alcohol use disorders and relapse for those who were previously abstinent. In terms of smoking, some studies find that people smoked more during the pandemic (Gendall et al., 2021; Guignard et al., 2021; Reynolds et al., 2021; Jackson et al., 2022). In contrast, others find that people were more likely to attempt to quit or successfully quit smoking (Jackson et al., 2021; Kayhan Tetik et al., 2021; Carreras et al., 2022).

Engaging in physical activity and maintaining healthy food-related behaviors are crucial for positive health outcomes. People may decrease their physical activity levels and increase sedentary time due to restrictions on public gatherings, closures of sports and entertainment facilities, and isolations and lockdowns (Naughton et al., 2021; Wedig et al., 2021). In contrast, people may increase their physical activity levels at home with a more flexible schedule during the pandemic (Ai et al., 2021; Mutz et al., 2021). The lockdown policies caused significant disruptions in food availability and accessibility (Niles et al., 2020; Ribeiro-Silva et al., 2021). A dramatic reduction in food expenditure was observed in rural areas or low- or middle-income countries (Mahmud and Riley, 2021; Wang et al., 2021). People who felt insecure about their job and food may shift their food consumption to ultra-processed foods, increasing the risk of weight gain (Adams et al., 2020; Pryor and Dietz, 2022).

Studies have demonstrated the association between job insecurity and poor mental health during the COVID-19 pandemic (Gong et al., 2022). Individuals losing their job during the pandemic were reported to have a higher risk of depression and anxiety than their employed peers in different countries, including the U.S. (Mcdowell et al., 2021), South Africa (Posel et al., 2021), and Spain (de Miquel et al., 2022). Bakkeli (2021) finds that worries and uncertainties regarding job security emerged as a significant predictor of lower life satisfaction during the COVID-19 pandemic. Overall, very few studies have investigated how job insecurity affected individuals' health and wellbeing during the COVID-19 pandemic and no such analysis for China.

We fulfill the gap in literature by investigating (a) the relationships between the perceived risk of unemployment and health outcomes (e.g., psychological wellbeing, physical health, and health behaviors) of Chinese adults; and (b) how the relationships differed between urban and rural adults.

## Data and key variables

This study uses the China Family Panel Studies (CFPS) data collected in 2018 (before the COVID-19 pandemic) and 2020^3^ (during the COVID-19 pandemic). The CFPS is a biennial longitudinal survey launched in 2010 by the Institute of Social Science Survey (ISSS) of Peking University, China (Xie and Hu, 2014). It collects a rich set of information from a nationally representative household sample in 31 provinces and autonomous regions/cities in China. This study uses the 2020 CFPS data for the analysis and leverages the 2018 CFPS data to control for the pre-pandemic socioeconomic status, psychological wellbeing, and health outcomes. We excluded the observations in Ningxia, Hainan, Qinghai, Inner Mongolia, and Tibet from the analysis as each province had <10 observations. We only keep observations who were 18–70 years in 2020. The total sample for the analysis is 9,308, including 6,925 rural adults and 2,383 urban adults.

This study also leverages the Oxford COVID-19 Government Response Tracker (OxCGRT)^4^ to control policy measures. OxCGRT created government policy indices for many countries, including China. We use two policy indicators, the stringency index and the economic support index. The stringency index measures the strictness of “lockdown style” policies that restrict individual behaviors and movements. The economic support index measures the supportive degree of economic policies, such as income support and debt relief. The 2020 CFPS participants were assigned the stringency index and economic support index based on their province of residence and the month when they completed their survey.

### Perceived unemployment risk

The variable of interest is the perceived risk of unemployment. Previous studies often use the state-level unemployment rate as a proxy for macroeconomic conditions (Ruhm, 2000, 2005; Janet et al., 2015; Triaca et al., 2020). We use the unemployment risk perceived by each individual to measure job insecurity during the COVID-19 pandemic for the following two main reasons. First, the state-level unemployment rate could capture neither individual heterogeneity on job security nor the effects of job insecurity on health outcomes, especially during the pandemic. For example, even among individuals who remained employed during the pandemic, there was significant perception of job insecure due to the increased occurrence of business closures and layoffs. This perception led to behavioral changes towards health shocks. Second, the official unemployment rate released by the Chinese government was likely to be underestimated as many rural-to-urban migrant workers would return home during the pandemic, and their employment status may not be appropriately recorded on time (Che et al., 2020). The 2020 CFPS participants were asked to answer the following question: “What is the probability you think that you will lose your job within the next 12 months as your companies may close down or lay off employees (0–100%)?” As shown in Table 1, the average perceived risk of unemployment risk was 20.8%; and it was statistically higher for rural adults (21.3%) than for urban adults (19.1%).

**Table 1:** Summary statistics of perceived unemployment risk and health wellbeing in both 2018 and 2020.

**Variable**	**Full sample**		**Rural sample**		**Urban sample**		**Mean difference^a^**
	**Mean**	**SD**	**Mean**	**SD**	**Mean**	**SD**	
**Variable of interest**							
Perceived risk of unemployment (0–1)	0.208	0.288	0.213	0.292	0.191	0.274	0.023^***^
**Health wellbeing in 2020**							
Life satisfaction (satisfied = 1, otherwise = 0)	0.691	0.462	0.685	0.464	0.708	0.455	−0.023^**^
Risk of depression	0.025	1.000	0.076	1.017	−0.124	0.932	0.200^***^
Self-rated health status (very good/excellent = 1, otherwise = 0)	0.320	0.467	0.333	0.471	0.283	0.451	0.049^***^
Weight gain from 2018 to 2020 (yes = 1, no = 0)	0.415	0.493	0.416	0.493	0.413	0.493	0.003
Obesity status (obese = 1, otherwise = 0)	0.093	0.290	0.086	0.280	0.114	0.318	−0.028^***^
Weekly frequency of physical activities	0.894	1.443	0.718	1.351	1.407	1.571	−0.689^***^
Daily digital screen time using mobile phones (hours)	2.008	2.694	1.692	2.486	2.924	3.043	−1.233^***^
Daily digital screen time using computers (hours)	0.641	1.842	0.409	1.529	1.315	2.417	−0.906^***^
Inadequate sleep (sleeping hours < 7 h = 1, otherwise = 0)	0.207	0.405	0.195	0.396	0.240	0.427	−0.044^***^
Smoking (daily number of cigarettes smoked)	4.653	8.419	4.868	8.631	4.028	7.739	0.840^***^
Drinking (weekly frequency ≥3 times = 1, otherwise = 0)	0.147	0.354	0.149	0.357	0.139	0.346	0.010
**Health wellbeing in 2018**							
Life satisfaction (satisfied = 1, otherwise = 0)	0.688	0.464	0.677	0.468	0.719	0.450	−0.042^***^
Risk of depression	−0.003	0.990	0.042	1.006	−0.136	0.928	0.179^***^
Self-rated health condition (very good/excellent = 1, otherwise = 0)	0.309	0.462	0.319	0.466	0.281	0.449	0.039^***^
BMI	23.32	3.395	23.23	3.381	23.59	3.423	−0.356^***^
Obesity status (obese = 1, otherwise = 0)	0.091	0.287	0.085	0.279	0.107	0.309	−0.021^***^
Weekly frequency of physical activity frequency	1.295	1.625	1.210	1.624	1.544	1.602	−0.334^***^
Daily digital screen time (hours)	1.264	1.690	1.107	1.602	1.722	1.849	−0.615^***^
Inadequate sleep (sleeping hours < 7 h = 1, otherwise = 0)	0.205	0.403	0.202	0.402	0.212	0.409	−0.010
Smoking (daily number of cigarettes smoked)	4.941	8.876	5.214	9.164	4.150	7.929	1.064^***^
Drinking (weekly frequency ≥3 times = 1, otherwise = 0)	0.166	0.372	0.171	0.376	0.151	0.359	0.019^**^
**No. of observations**	9,308		6,925		2,383		

a “Mean difference” represents the mean comparison of each variable between rural and rural adults based on t-tests.

* p < 0.1.

** p < 0.05.

*** p < 0.01.

### Health status: psychological wellbeing and physical health

We use both life satisfaction and depression risk to measure the psychological wellbeing of adults. The 2020 CFPS participants were asked to answer the following question: “How do you rate your life satisfaction on a scale from one (not satisfied at all) to five (extremely satisfied)?” We create a binary variable for life satisfaction that equals one if the participant chose a scale of 4 or 5; and zero otherwise. The CFPS adopted eight questions according to the Center for Epidemiology Studies Depression (CES-D scale) (Radloff, 1977). The CFPS provides a depression scale by summing the answer to these eight questions, where each question scores from one to four. We standardize the depression scale to have a mean of zero and a standard deviation of one. A higher value of the depression risk suggests a higher probability of being depressed.

Based on the available information in the CFPS, we use weight status and self-rated health status to measure physical health. The participant's body mass index (BMI) is calculated based on their self-reported weight and height. A binary variable is created to indicate obesity status: it equals one if BMI is equal to or greater than 28; and zero otherwise^5^ (Pan et al., 2021). We also create the other binary variable indicating whether the participants gained weight in 2020 compared with 2018. The participants were also asked to rate their health status on a scale from one (excellent health) to five (poor health). A binary variable is created to indicate an excellent or very good health status if a scale of one or two was chosen.

Table 1 presents summary statistics of the key dependent variables measuring health outcomes for all the sample, rural adults, and urban adults during the pandemic (2020) and before the pandemic (2018), respectively. Compared with urban adults, rural adults had significantly lower life satisfaction (0.685 vs. 0.708), a higher depression risk (0.076 vs. −0.124), a significantly higher probability of having self-rated very good-to-excellent health condition (0.333 vs. 0.283), and a lower obesity risk (0.086 vs. 0.114). No statistically significant difference was found in the probability of gaining weight between rural and urban adults. Qualitatively similar rural–urban differences were found for psychological wellbeing and physical health in 2018.

### Health behaviors

We investigate both health-promising activities (e.g., physical activity) and health-compromising behaviors (e.g., inadequate sleep, screen time, and substance use). The CFPS participants were asked to answer the following question: “How often do you participate in physical activities in the last 12 months, excluding walking or biking to work?” We create a categorical variable to measure the weekly frequency of physical activities. It would equal zero if they chose “less than one time per week,” one for “1–2 times per week,” two for “3–4 times per week,” three for “5–6 per week,” and four for “seven times or above per week.” The 2020 CFPS participants were also asked to separately report their daily digital online hours using either mobile devices or computers in 2020, while the 2018 participants were asked to report their total weekly digital online hours for leisure using either mobile devices or computers. We create the daily digital online screen time in 2018 to control sedentary behavior before the pandemic. We also created variables measuring digital time using mobile phones and computers in 2020 separately. The participants were asked to report their sleeping hours on a typical day.^6^ Blanchflower and Bryson (2021) reported that a higher unemployment rate is associated with a higher probability of lacking sleep (<7 h). We create a binary variable indicating inadequate sleep if the participant reported sleeping <7 h on a typical day. The participants were also asked to answer two questions about substance use: “How many cigarettes did you consume daily in the last month?” and “Did you drink at least three times per week in the last month?” The number of cigarettes smoked daily in the last month is used as one of the outcome variables. We also create a binary variable indicating whether drinking at least three times per week in the last month.

As shown in Table 1, compared with the pre-pandemic level, the CFPS participants had a much lower frequency of physical activities and longer digital screen time during the pandemic. Digital screen time using mobile phones was much longer than digital screen time using computers in 2020: 2.008 hours vs. 0.641 hours per day. Furthermore, compared with urban adults, rural adults had a significantly lower frequency of physical activities (0.718 vs. 1.407), shorter digital screen time using mobile phones (1.692 hours vs. 2.924 hours per day) and computers (0.409 hours vs. 1.315 hours per day), a smaller probability of inadequate sleep (0.195 vs. 0.240), and more cigarettes smoked daily (4.868 vs. 4.028). No statistical difference in drinking behavior was found between rural and urban adults in 2020.

### Control variables

We control for several sets of factors in the regression analyses. First, we control for health outcomes and psychological wellbeing in the pre-pandemic period using the 2018 CFPS data. Specifically, we include annual household income (10,000 Yuan), household net assets (10,000 Yuan), total liquid assets consisting of bank savings and brokerage accounts (10,000 Yuan), and whether at least one family member was a migrant worker in 2018. These covariates reflect economic/financial status and migrants' experience before the pandemic. Second, we control for personal, household, and regional characteristics in 2020. Personal characteristics include age, age squared, and sex, belonging to the Han ethnic group or not, education level, marital status, ever being diagnosed with any chronic diseases or not, and self-rated interpersonal relationship on a scale from 1 (extremely poor) to 10 (extremely good). We also include two labor market outcome variables, a dummy variable indicating the major job type (non-agricultural vs. agricultural), and whether the participant was self-employed in non-agricultural sectors. We also include economic and financial variables: whether covered by medical insurance or not, whether having retirement plans, personal income in the past 12 months (10,000 Yuan)^7^, and self-rated relative income status (below, around, and above the average in one's neighborhood). Household characteristics consist of the number of children under 16 years and the number of family members. Regional characteristics include self-rated neighborhood medical services and the monthly average of new COVID cases at the province level in the last 3 months before the interview month for each observation. We also control for two OxCGRT policy indicators at the province level: the stringency index and the economic support index, in the month when the CFPS participant was interviewed. Province-fixed effects are also included.

Table 2 shows that the CFPS participants were 44.10 years on average, more males (55.5%) than females, and the majority belonged to the Han ethnic group (92.5%) and were married (84.2%). About one-third had up to 6 years of schooling (elementary school), one-third completed junior high school, 16.5% completed high school, and 17.1% received associated degrees or above. About 12.7% of the participants had been diagnosed with chronic diseases. About 60.7% worked mainly in the non-agricultural sectors, and 10.6% were self-employed in non-agricultural business. The participants had an average score of 7.08 in a 10-point scale for the self-rated interpersonal relationship. Before the pandemic, on average, the participant households had ~100,200 Yuan for annual household income, 773,000 Yuan for household net assets, and 71,520 Yuan for household liquid assets. During the pandemic, the average personal annual income was 39,310 Yuan; 63.6% had retirement plans; and 91.4% had medical insurance. At the regional level, 60.9% of the participants considered their neighborhood medical services satisfactory; the monthly new COVID cases reported in the residing province was 1.236; the monthly stringency index and economic support index at the province level were 49.48 and 37.62 in a 100-point scale in 2020.

**Table 2:** Summary statistics of control variables.

**Variable**	**Surveyed year**	**Full sample**		**Rural sample**		**Urban sample**		**Mean difference^a^**
		**Mean**	**SD**	**Mean**	**SD**	**Mean**	**SD**	
**Personal characteristics**								
Age	2020	44.10	12.63	44.73	12.95	42.27	11.47	2.455^***^
Age squared	2020	2,104	1,134	2,168	1,167	1,918	1,010	249.8^***^
Male (male = 1, female = 0)	2020	0.555	0.497	0.558	0.497	0.547	0.498	0.011
Ethnicity (Han = 1, otherwise = 0)	2020	0.925	0.263	0.917	0.276	0.950	0.219	−0.033^***^
Highest education level	2020							
At most elementary schools (yes = 1, no = 0)		0.333	0.471	0.332	0.471	0.337	0.473	−0.006
Completed junior high school (yes = 1, no = 0)		0.330	0.470	0.328	0.469	0.337	0.473	−0.009
Completed high school (yes = 1, no = 0)		0.165	0.371	0.169	0.375	0.154	0.361	0.016^*^
Had an associate degree or above (yes = 1, no = 0)		0.171	0.377	0.171	0.377	0.172	0.378	−0.001
Marital status (marriage = 1, otherwise = 0)	2020	0.842	0.365	0.847	0.360	0.827	0.378	0.020^**^
Diagnosed with chronic diseases (yes = 1, no = 0)	2020	0.127	0.333	0.130	0.337	0.118	0.323	0.0120
Self-employed in non-agricultural business (yes = 1, no = 0)	2020	0.106	0.308	0.102	0.302	0.120	0.325	−0.019^**^
Major job type (non-agricultural = 1, agricultural = 0)	2020	0.607	0.488	0.515	0.500	0.874	0.332	−0.359^***^
Having retirement plans (yes = 1, no = 0)	2020	0.636	0.481	0.613	0.487	0.703	0.457	−0.090^***^
Having medical insurance (yes = 1, no = 0)	2020	0.914	0.281	0.915	0.279	0.910	0.287	0.005
Self-rated interpersonal relationship scale (1–10)	2020	7.081	1.816	7.102	1.874	7.021	1.636	0.082^*^
Personal income in the last 12 months (10,000 Yuan)	2020	3.931	3.694	3.347	2.787	5.629	5.183	−2.282^***^
Self-rated income status in the neighborhood	2020							
Below average		0.273	0.446	0.274	0.446	0.271	0.444	0.004
Around average		0.519	0.500	0.501	0.500	0.569	0.495	−0.068^***^
Above average		0.208	0.406	0.224	0.417	0.160	0.367	0.064^***^
**Family characteristics**								
No. of children under 16 years	2020	1.310	1.357	1.406	1.392	1.031	1.207	0.375^***^
No. of household members	2020	4.175	2.009	4.295	2.067	3.828	1.787	0.467^***^
Annual household income (10,000 Yuan)	2018	10.02	17.17	8.22	11.32	15.24	27.25	−7.025^***^
Net assets (10,000 Yuan)	2018	77.30	170.0	53.68	139.6	145.9	223.6	−92.26^***^
Liquid assets (10,000 Yuan)	2018	7.152	20.885	4.565	13.641	14.670	32.976	−10.11^***^
Family members ever being migrant workers (yes = 1, no = 0)	2018	0.462	0.499	0.537	0.499	0.244	0.430	0.293^***^
**Regional characteristics**								
Perception of neighborhood medical services (good/very good = 1, otherwise = 0)	2020	0.609	0.488	0.595	0.491	0.650	0.477	−0.056^***^
Monthly new COVID cases at the province level	2020	1.236	2.774	1.001	2.434	1.921	3.494	−0.920^***^
Monthly stringency index at the province level	2020	49.48	8.137	48.68	8.038	51.81	7.973	−3.137^***^
Monthly economic support index at the province level	2020	37.62	13.18	38.21	13.41	35.92	12.34	2.290^***^
No. of observations		9,308		6,925		2,383		

^a“^Mean difference” represents the mean comparison of each variable between rural and urban adults by t-tests.

* p < 0.1.

** p < 0.05.

*** p < 0.01.

## Estimation methodology

We examine the relationships between the perceived risk of unemployment and the psychological wellbeing, health status, and health behaviors of the CFPS participants controlling for confounding factors at the personal, household, and regional levels. Ordinary linear regression (OLS) models or logit models are employed depending on the nature (continuous vs. discrete) of the dependent variables.


(1) $$\begin{array}{r} H_{ip}^{2020}=\alpha +\beta Unemployment_{ip}+\gamma E_{ip}^{2018}+\delta X_{ip}^{2020}+\theta H_{ip}^{2018}\\ +\lambda_{p}+\mu_{ip} \end{array}$$

The variable of interest, *unemployment*_*ip*_, indicates the perceived risk of unemployment in 2020 by individual *i* residing in province *p*. The dependent variable, denoted by $H_{ip}^{2020}$, is one of the health outcomes. Due to the concern of potential serial correlations, we control for health outcomes in 2018 indicated by $H_{ip}^{2018}$. We also control for the family's financial and economic status in 2018, denoted by $E_{ip}^{2018}$, including annual household income, net household assets, household liquid assets, and whether ever having a migrant worker in the family. Those variables reflect family financial strength and migrating experience, and they are expected to affect health outcomes in 2020. $X_{ip}^{2020}\;$is a vector of control variables representing personal, household, and regional characteristics in 2020. Equation (1) also includes province-fixed effects denoted by λ_*p*_ and error term denoted by μ_*ip*_.

## Results

We present the OLS coeffects of key independent variables for health outcomes measured by a continuous variable (i.e., depression risk, weekly frequency of physical activities, digital screen time using mobile devices or computers, and daily number of cigarettes smoked). For the health outcomes measured by a binary variable, including life satisfaction, self-rated very good-to-excellent health condition, weight gain, obesity status, having inadequate sleep, and drinking at least three times per week in the last month, we present the marginal effects of key independent variables based on the corresponding logit model estimation.

### Psychological wellbeing

We first investigate the relationship between the perceived risk of unemployment and psychological wellbeing (e.g., life satisfaction and depression risk) during the COVID-19 pandemic and summarize the results in Table 3. For the national sample, the perceived risk of unemployment was significantly associated with life satisfaction (Column 1) and depression risk (Column 4). A higher unemployment risk was found to be associated with lower life satisfaction and an escalated risk of depression. Table 3 also shows rural–urban variations — the association between the perceived unemployment risk and depression risk was statistically significant for both rural and urban adults and greater in magnitude for rural adults than for urban adults. On the other hand, the unemployment risk was only significantly associated with decreased life satisfaction for rural adults (*P* <1%), but no statistically significant association was found for urban adults. The results indicate that compared with urban adults, rural adults were more vulnerable to unemployment risk and exposed to greater adverse impacts on their psychological wellbeing during the pandemic.

**Table 3:** Regression results on the associations between the perceived risk of unemployment and psychological wellbeing.

	**Life satisfaction**			**Depression risk**		
	**Full sample**	**Rural**	**Urban**	**Full sample**	**Rural**	**Urban**
	**(1)**	**(2)**	**(3)**	**(4)**	**(5)**	**(6)**
Perceived unemployment risk	−0.316^***^ (0.090)	−0.330^***^ (0.103)	−0.257 (0.191)	0.305^***^ (0.031)	0.325^***^ (0.037)	0.243^***^ (0.062)
Life satisfaction in 2018	1.205^***^ (0.053)	1.133^***^ (0.061)	1.444^***^ (0.110)			
Depression risk in 2018				0.434^***^ (0.009)	0.437^***^ (0.011)	0.416^***^ (0.019)
*R* ^2^				0.286	0.287	0.288
Pseudo *R*^2^	0.202	0.205	0.212			
Control	Y	Y	Y	Y	Y	Y
No. of observations	9,308	6,925	2,383	9,308	6,925	2,383

This table presents the estimated coefficients of the perceived unemployment risk on life satisfaction and the risk of depression. Standard errors are shown in parentheses.

* p < 0.1.

** p < 0.05.

*** p < 0.01.

The control variables at the individual level in 2020 are age, age squared, gender, belonging to the Han ethnic group or not, education level, marital status, ever being diagnosed with chronic diseases or not, being self-employed in non-agricultural sectors or not, major job type (non-agricultural vs. other), covered by medical insurance or not, having retirement plans or not, self-rated interpersonal relationship scale, personal annual income (10,000 Yuan), and self-rated income status. We also control for the following family characteristics: the number of children under 16 years and the number of household members in 2020 as well as annual household income (10,000 Yuan), net assets (10,000 Yuan), liquid financial assets (10,000 Yuan), and life satisfaction (Columns 1–3) and depression risk (Columns 4–6) in 2018. The regional characteristics we control for are the perceived neighborhood medical services, the number of monthly new COVID cases, and the monthly stringency index and economic support index at the province level. All regressions include the province-fixed effects.

### Physical health status

We further investigate the effects of the perceived unemployment risk on the self-reported physical health status and summarize the results in Table 4. The perceived risk of unemployment was only negatively associated with the self-rated very-good-to-excellent health condition for urban adults, and the associations stayed negative but statistically insignificant for the national sample and rural adults (Columns 1–3). The differences in the relationship between unemployment risk and self-rated physical health could potentially attributed to the urban–rural differences in their awareness of physical health. Urban adults might be more health cautious and sensitive to their physical health than rural adults, while rural adults might have lower healthcare utilization (Ying et al., 2020).

**Table 4:** Regression results on the associations between the perceived risk of unemployment and physical health.

	**Self-rated health condition**			**Weight gain**			**Obesity**		
	**Full sample**	**Rural**	**Urban**	**Full sample**	**Rural**	**Urban**	**Full sample**	**Rural**	**Urban**
	**(1)**	**(2)**	**(3)**	**(4)**	**(5)**	**(6)**	**(7)**	**(8)**	**(9)**
Perceived unemployment risk	−0.130 (0.090)	−0.093 (0.102)	−0.356^*^ (0.200)	−0.180^**^ (0.078)	−0.210^**^ (0.089)	−0.064 (0.165)	−0.426^**^ (0.179)	−0.454^**^ (0.211)	−0.497 (0.356)
Self-rated health status in 2018	1.560^***^ (0.052)	1.534^***^ (0.060)	1.675^***^ (0.109)						
BMI in 2018				−0.137^***^ (0.007)	−0.136^***^ (0.008)	−0.147^***^ (0.015)			
Obesity status in 2018							4.327^***^ (0.104)	4.431^***^ (0.126)	4.276^***^ (0.200)
Pseudo *R*^2^	0.174	0.175	0.183	0.0422	0.0430	0.0581	0.433	0.441	0.437
Control	Y	Y	Y	Y	Y	Y	Y	Y	Y
No. of observations	9,308	6,925	2,383	9,308	6,925	2,383	9,308	6,861	2,349

This table presents the estimated coefficients of the perceived unemployment risk on physical health. Standard errors are shown in parentheses.

* p < 0.1.

** p < 0.05.

*** p < 0.01.

We include self-rated health condition in 2018 (Columns 1–3), BMI in 2018 (Columns 4–6), and obesity status in 2018 (Columns 7–9) as additional covariates. The other covariates are the same as those in Table 3. All regressions include the province-fixed effects.

Obesity is a public epidemic in high-income countries and has become a rising public health issue for low-income countries (Janet et al., 2015; Triaca et al., 2020). We examine the association between the perceived risk of unemployment and the following two weight measures: whether gained weight in 2020 compared to the 2018 level and whether being obese or not in 2020. Columns (4)–(9) show that a higher perceived risk of unemployment was statistically associated with a lower probability of gaining weight or being obese for the full sample and rural adults (*P* < 5%), but no statistically significant associations were found for urban adults.

Overall, the results show the rural–urban differences in the relationships between the perceived risk of unemployment and physical health outcomes. A higher perceived risk of unemployment was significantly associated with poor self-reported physical health for urban adults, but the relationship was statistically insignificant for rural adults. On the other hand, a higher unemployment risk was significantly associated with a lower probability of gaining weight and being obese for rural adults; but the relationships were statistically insignificant for urban adults.

### Health behaviors

Health-promising and compromising behaviors are important factors in the health production function (Grossman, 1972). Based on literature and available information on CFPS, we examine health-promising behaviors such as physical activity and health-compromising behaviors such as inadequate sleep, substance use, and digital screen time.^8^

Three indicators are used to measure physical activities: (1) weekly frequency of physical activities in the last 12 months, (2) daily hours spent on screen time using mobile phones, and (3) daily hours spent on screen time using computers. Table 1 shows that digital screen time using mobile phones was more predominant than that using computers for Chinese adults. The previous studies document that Chinese mobile users spent more time on digital screen time using mobile phones during the pandemic than the pre-pandemic level: 5.7 h in 2020 vs. 4.3 h in 2018 (Thomala, 2022), as people would spend more time to search information through the Internet when facing uncertainties (Fang et al., 2019). As shown in Table 5, no statistically significant relationships were found between the perceived risk of unemployment and the weekly frequency of physical activities (Columns 1–3) and digital screen time using mobile phones (Columns 4–6) for both rural and urban adults. The perceived risk of unemployment was negatively and significantly associated with digital screen time using computers for rural adults, but the association was statistically insignificant for urban adults (Columns 7–9).

**Table 5:** Regression results on the associations between the perceived risk of unemployment and physical activity and screen time.

	**Weekly frequency of physical activities**			**Digital online screen time**					
				**Using mobile phones**			**Using computers**		
	**Full sample**	**Rural**	**Urban**	**Full sample**	**Rural**	**Urban**	**Full sample**	**Rural**	**Urban**
	**(1)**	**(2)**	**(3)**	**(4)**	**(5)**	**(6)**	**(4)**	**(5)**	**(6)**
Perceived unemployment risk	−0.071 (0.050)	−0.061 (0.055)	−0.011 (0.113)	−0.104 (0.079)	−0.113 (0.084)	−0.030 (0.198)	−0.164^***^ (0.060)	−0.140^**^ (0.058)	−0.130 (0.168)
Weekly frequency of physical activities in 2018	0.213^***^ (0.009)	0.186^***^ (0.010)	0.276^***^ (0.020)						
Digital online screen time using mobile phones and computers in 2018				0.401^***^ (0.015)	0.360^***^ (0.018)	0.475^***^ (0.031)	0.087^***^ (0.011)	0.038^***^ (0.012)	0.174^***^ (0.026)
*R* ^2^	0.140	0.092	0.163	0.367	0.366	0.312	0.235	0.214	0.216
Control	Y	Y	Y	Y	Y	Y	Y	Y	Y
No. of observations	9,308	6,925	2,383	9,308	6,925	2,383	9,308	6,925	2,383

This table presents the estimated coefficients of the perceived unemployment risk on physical activity patterns. Standard errors are shown in parentheses.

* p < 0.1.

** p < 0.05.

*** p < 0.01.

We include weekly frequency of physical activities (Columns 1–3) and daily digital online screen time using mobile phones and computers in 2018 (Columns 4–9) as additional covariates. The other covariates are the same as those in Table 3. All regressions include the province-fixed effects.

The rural–urban variations of the associations between the perceived unemployment risk and inadequate sleep or substance use were quite intriguing (Table 6). A higher perceived risk of unemployment was significantly associated with an increased probability of inadequate sleep (<7 h per day) for the full sample (*P* < 10%) and rural adults (*P* < 1%), while such a relationship was statistically insignificant for urban adults. On the other hand, the perceived unemployment risk played a significant and negative role in smoking and drinking behaviors for urban adults, but not for rural adults. In China, smoking and drinking are more prevalent among males than females. The CFPS2020 data show that the average number of cigarettes smoked daily was 8.24 for males and only 0.17 for females; and the percentage for drinking at least three times a week was 24.61% for males and only 2.30% for females. We re-estimate the results for males only and present the results in Table A1. The results for male adults were qualitatively similar to the main results—the associations between the perceived risk of unemployment and substance use were statistically significant and negative for urban male adults but statistically insignificant for rural male adults.

**Table 6:** Regression results on the associations between the perceived risk of unemployment and inadequate sleep and substance use.

	**Inadequate sleep**			**Substance use**					
				**Smoking**			**Drinking**		
	**Full sample**	**Rural**	**Urban**	**Full sample**	**Rural**	**Urban**	**Full sample**	**Rural**	**Urban**
	**(1)**	**(2)**	**(3)**	**(4)**	**(5)**	**(6)**	**(4)**	**(5)**	**(6)**
Perceived unemployment risk	0.183^*^ (0.094)	0.287^***^ (0.109)	−0.170 (0.191)	−0.078 (0.179)	0.127 (0.218)	−0.605^**^ (0.287)	−0.070 (0.128)	0.019 (0.145)	−0.505^*^ (0.284)
Inadequate sleep in 2018	1.315^***^ (0.059)	1.278^***^ (0.069)	1.441^***^ (0.113)						
No. of cigarettes smoked daily in 2018				0.715^***^ (0.007)	0.686^***^ (0.008)	0.818^***^ (0.011)			
Drink at least three times weekly in 2018							2.348^***^ (0.075)	2.274^***^ (0.087)	2.669^***^ (0.159)
*R* ^2^				0.671	0.647	0.777			
Pseudo *R*^2^	0.0927	0.0938	0.103				0.335	0.342	0.352
Control	Y	Y	Y	Y	Y	Y	Y	Y	Y
No. of observations	9,308	6,925	2,383	9,308	6,925	2,383	9,308	6,925	2,378

This table presents the estimated coefficients of the perceived unemployment risk on inadequate sleep and substance use. Standard errors are shown in parentheses.

* p < 0.1.

** p < 0.05.

*** p < 0.01.

We include whether having inadequate sleep in 2018 (Columns 1–3), the daily number of cigarettes smoked in 2018 (Columns 4–6), and whether drinking at least three times per week in 2018 (Columns 7–9) as additional covariates. The other covariates are the same as those in Table 3. All regressions include the province-fixed effects.

## Discussion

This study contributes to the ongoing discussion on the relationships between economic conditions and health outcomes by uncovering the associations between the perceived risk of unemployment and a broad spectrum of health outcomes for Chinese adults during the COVID-19 pandemic. Our descriptive statistics in Table 1 show that urban adults had a lower risk of depression than rural adults, which is consistent with previous studies (Zhang et al., 2012, 2022). We find that rural adults in China were more vulnerable than urban adults when they perceived their jobs as insecure. The perceived risk of unemployment was significantly and negatively associated with depression risk, and the correlation was greater for rural adults than for urban adults. The association between unemployment risk and life satisfaction was statistically significant and negative for rural adults but insignificant for urban adults. Our findings, to a great extent, are consistent with recent studies showing that the fear of COVID-19 infection, job insecurity, and loss of employment income were associated with aggravated life satisfaction during the pandemic (Bakkeli, 2021; Mahmud and Riley, 2021; Satici et al., 2021). Our results can be potentially explained by the much higher unemployment rate for rural migrant workers than for urban workers as well as for people with lower education levels or unprofessional skills (Che et al., 2020). Although the Chinese government has implemented various economic stimulus policies during the pandemic, a few of them specifically targeted the rural population (Wang et al., 2021). One policy implication of our findings is to call for more economic stimulus policies to improve job opportunities for rural adults to improve their wellbeing.

We also find that a higher perceived risk of unemployment was significantly associated with a lower probability of having better self-rated health for urban adults (*P* < 10%), but not for rural adults. Several factors can potentially explain this. First, urban adults in China in general are more health cautious than rural adults, which is partly reflected by much lower self-reported very-good-to-excellent health status for urban adults than rural adults (see Table 1). Second, rural and urban adults generally have different living environments. The living environment for urban residents is more populated and denser, while rural residents have larger living spaces augmented by their indoor spaces, yards, and farms. Although non-farm activities were restricted during the lockdown period for urban adults, rural adults could still maintain some of their agricultural production activities. Therefore, rural adults were likely to keep their routine farm work or physical activities, even though their non-farm work was unsecured.

An increasing number of empirical studies offer mixed results on the relationship between economic conditions and weight-related health outcomes (Cawley, 2015). Ruhm (2000) used Behavioral Risk Factor Surveillance System (BRFSS) data from 1987 to 1995 and found that an increase in the state unemployment rate in the U.S. was associated with reduced obesity, increased physical activities, and improved diet (measured by daily grams of fat consumed). Similarly, a decrease in employment rates was found to reduce the risk of obesity, especially severe obesity based on the BRFSS data in 1987–2000 (Ruhm, 2005). Triaca et al. (2020) also report similar results for Brazilian pollution in 2006–2014: an increase in unemployment rates significantly reduced the probability of being overweight, obese, and severely obese. However, positive associations between the unemployment rate and weight measures were reported in a Finnish cohort (Böckerman et al., 2007) and among African-American men in metropolitan statistical areas in the U.S. (Charles and DeCicca, 2008). The divergent findings suggest considerable heterogeneity in the effects of unemployment on weight-related health outcomes across gender, region, urbanicity, and socioeconomic status (Cawley, 2015). This study expands this growing literature by exploring rural and urban variations on the associations between the perceived risk of unemployment and weight measures during the COVID-19 pandemic. We find that rural adults in China were less likely to gain weight and be obese if they perceived their job as insecure, while the association was not statistically significant for urban adults.

The relationships between unemployment risk and health behaviors found in this study are quite interesting. Ruhm (2000) reports increased physical activities as the unemployment rate went up in the business fluctuation cycles. One possible reason was that decreased opportunity cost of leisure time during economic downturns would encourage people to spend more time in physical activities. However, this study finds no statistically significant relationship between unemployment risk and physical activities for both rural and urban adults in China. The insignificant relationships could result from the lockdown policies during the pandemic: urban adults may face restricted access to sports/entertainment facilities. An Uganda study found that rural households spent more time on agricultural farming or livestock production to make up for the decline or even loss of non-farm income during the COVID-19 lockdown (Mahmud and Riley, 2021). Similarly, rural Chinese adults, especially rural-to-urban migrants, would increase their labor supply to farm work, if they perceived their non-agricultural job unsecured, declined, or even lost. This could be a possible explanation for the statistically insignificant association between job insecurity and physical activities for rural adults, even though the opportunity cost of time was reduced.

The heterogeneous associations between unemployment risk and health behaviors between rural and urban adults suggest that they might have responded to or coped with the pandemic-driven unemployment risk differently. The perceived risk of unemployment was significantly and positively associated with inadequate sleep for rural adults, but the relationship was statistically insignificant for urban adults. Unemployment risk was statistically and negatively associated with substance use for urban adults, but the relationships were statistically insignificant for rural adults. Our finding on inadequate sleep for rural adults is similar to the finding in Blanchflower and Bryson (2021), where an increased unemployment rate was reported to associate with poor sleep quality for the U.S. and European populations. Inadequate sleep was also found to significantly correlate with the increased risk of depression (Zhai et al., 2015). Our finding on inadequate sleep for rural adults is also consistent with what we found on psychological wellbeing: rural adults who perceived an increased risk of unemployment were more likely to have inadequate sleep, experience depression, and have lower life satisfaction.

Previous studies have found that consumption of alcohol and cigarettes increased due to mental stress in economic downturns despite decreased income (Charles and DeCicca, 2008; De and Segura-Escano, 2021; Alam and Bose, 2022). Our results are quite different in the context of the COVID-19 pandemic. Health concerns could be one of the reasons to explain such a difference. The WHO has stated that tobacco smoking^9^ and alcohol disorders^10^ would put people at high risk of developing severe COVID-19 symptoms. Urban adults in China might reduce their smoking and drinking due to health concerns, though job insecurity might impose significant pressure on them. Another explanation for decreased smoking is the concern about second-hand smoking to their family members during the COVID-19 lockdown (Cai and Zhou, 2022). Such concern might not play a significant role in rural adults as they had greater living spaces and more open areas (yards and farms). Furthermore, the concern of job insecurity along with lockdown policies might reduce business gatherings for the social network, where alcohol and cigarette consumption frequently occurred. It could be another possible explanation for reduced substance use for urban adults during the pandemic.

There are several limitations in this study. First, we face measurement bias as job insecurity and health outcomes were self-reported. Second, although we control for the pre-pandemic economic and health status in 2018 as well as policy measures upon the COVID-19 pandemic in 2020, our empirical analyses were based on the cross-sectional data, making it challenging to pin down the causal effects of job insecurity on health outcomes. Therefore, this study is likely to be plagued by omitting variable problems and/or collinearity between job insecurity and the control variables. Third, we only investigate the short-term effects of the perceived unemployment risk on health outcomes and behaviors, as the latest released CFPS data were collected from July to December 2020. Chinese government kept the stringent mobility restrictions and lockdown policies, also known as the “zero-COVID” policy, until the end of 2022. Future investigations are merited when China removed the “zero-COVID” policy in early 2023.

We envision future research in several directions. First, we focus on the rural–urban variation as we were inspired by the predominant rural–urban disparities in various socioeconomic conditions in China. It would be interesting to investigate the differences in the relationships between job insecurity and health outcomes by other socioeconomic factors. For example, given that females and males have different attitudes toward economic uncertainties and responsibilities within families (Fang et al., 2020; Shepherd, 2022), they would have different psychological and behavioral responses (e.g., health outcomes and parenting styles) to unemployment risk. Second, we provide a comprehensive picture of the associations between job insecurity and health outcome and behaviors for rural and urban adults in China during the COVID-19 pandemic. Yet, the study cannot address the causal relationship and offer explorations on potential mechanisms due to the lack of data. Future research is merited to explore the potential causal relationship between job insecurity and health outcome and mechanisms through which job insecurity can affect health outcomes during the COVID-19 pandemic when data become available.

## Conclusion

This study investigates the associations between the perceived risk of unemployment and psychological wellbeing, physical health, and health behaviors among Chinese adults during the COVID-19 pandemic and explores the rural–urban variation in such associations. We find that a higher perceived risk of unemployment was statistically associated with lower depression risk and aggravated life satisfaction, and such correlation was greater for rural adults than urban adults. A negative and statistically significant association was observed between the perceived unemployment risk and self-rated health conditions for urban adults, but the relationship was statistically insignificant for rural adults. Rural adults who perceive their job as insecure were more likely to have inadequate sleep, reduce compute-screen time, and less likely to gain weight and be obese. We also find that the perceived unemployment risk was significantly and negatively associated with health-compromising behaviors (smoking and drinking) for urban adults, but not for rural adults. These findings imply that rural and urban adults had different psychological and behavioral responses to the unemployment risk that was tightly related to the pandemic. Public policies aimed to improve health and employment should be designed from different angles targeting urban and rural populations.

## Data availability statement

The datasets presented in this study can be found in online repositories. The names of the repository/repositories and accession number(s) can be found at: CFPS official website: https://www.isss.pku.edu.cn/cfps/en/index.htm.

## Ethics statement

The studies involving human participants were reviewed and approved by Peking Univeristy. The patients/participants provided their written informed consent to participate in this study.

## Author contributions

FZ: conceptualization, funding acquisition, writing—reviewing and editing, and supervision. HX: data analysis, validation, and writing—original draft. YJ: supervision, methodology, and writing—reviewing and editing. MZ: data analysis, writing—original draft, and writing—reviewing and editing. All authors contributed to the manuscript and approved the submitted version.

## Footnotes

^1^The GDP growth rate in China from 2011 to 2020 is 9.55%, 7.86%, 7.77%, 7.43%, 7.04%, 6.85%, 6.95%, 6.75%, 5.95%, and 2.24%, respectively. https://data.stats.gov.cn/easyquery.htm?cn=C01. Last access was on 14 February 2023.

^2^The annual unemployment rates in urban China from 2011 to 2020 are 4.1%, 4.1%, 4.1%, 4.1%, 4.1%, 4.0%, 3.9%, 3.8%, 3.6%, and 4.2%, respectively. https://data.stats.gov.cn/easyquery.htm?cn=C01. Last access was on 14 February 2023.

^3^The 2020 CFPS was conducted from July to December 2020. Due to the lockdowns and mobility restrictions, about 88.5% of the participants were surveyed by telephone.

^4^OxCGRT has collected systemic information on government policy measures to tackle COVID-19 worldwide. Detailed information on data construction and codebook can be found at https://www.bsg.ox.ac.uk/research/COVID-19-government-response-tracker. Last access was on 10 February 2023.

^5^According to the working group on obesity and health industry standard (WS/T 428-2013) in China, Chinese people with BMI equal to or greater than 28.0 kg/m^2^ are defined as obese (Pan et al., 2021).

^6^We excluded the CPFS participants who reported more than 15 h of sleep a day.

^7^Ideally, we would like to control household income in 2020 as it is one of the most important factors for health wellbeing. Unfortunately, CFPS has not released the data on household income and wealth in 2020.

^8^Dietary behavior is also a critical lifestyle component and affects health status. Unfortunately, the 2020 CFPS does not collect detailed dietary-related information.

^9^https://www.who.int/news/item/11-05-2020-who-statement-tobacco-use-and-COVID-19. Last accessed on 6 February 2023.

^10^https://www.euro.who.int/__data/assets/pdf_file/0010/437608/Alcohol-and-COVID-19-what-you-need-to-know.pdf. Last accessed on 6 February 2023.

## Appendix

**Table A1:** Regression results on the associations between the perceived risk of unemployment and substance use for males only.

	**Substance use**					
	**Smoking**			**Drinking**		
	**Full sample for male adults**	**Rural male sample**	**Urban male sample**	**Full sample for male adults**	**Rural male sample**	**Urban male sample**
	**(1)**	**(2)**	**(3)**	**(4)**	**(5)**	**(6)**
Perceived unemployment risk	–0.145 (0.316)	0.261 (0.383)	–1.165^**^ (0.520)	–0.014 (0.018)	0.001 (0.021)	–0.071^*^ (0.038)
No. of cigarettes smoked daily in 2018	0.714^***^ (0.009)	0.684^***^ (0.011)	0.819^***^ (0.015)			
Drink at least 3 times weekly in 2018				0.304^***^ (0.007)	0.300^***^ (0.008)	0.314^***^ (0.014)
*R* ^2^	0.576	0.543	0.727			
Pseudo *R*^2^				0.233	0.236	0.271
Control	Y	Y	Y	Y	Y	Y
No. of observations	5,169	3,865	1,304	5,169	3,865	1,302

This table presents the estimated coefficients (margin effect) of the perceived unemployment risk on substance use just for the male sample. Standard errors are shown in parentheses.

* p < 0.1.

** p < 0.05.

*** p < 0.01.

We include whether having the daily number of cigarettes smoked in 2018 (Columns 1–3), and whether drinking at least three times per week in 2018 (Columns 4–6) as additional covariates. The other covariates are the same as those in Table 3. All regressions include the province-fixed effects.
